# Safety and efficacy of coronavirus disease‐19 vaccines in chronic kidney disease patients under maintenance hemodialysis: A systematic review

**DOI:** 10.1002/hsr2.700

**Published:** 2022-06-16

**Authors:** Neha Mehta, Sangam Shah, Kiran Paudel, Rajan Chamlagain, Santosh Chhetri

**Affiliations:** ^1^ Tribhuvan University Teaching Hospital Maharajgunj Nepal; ^2^ Division of Research Affairs, Larkins Community Hospital South Miami Florida USA; ^3^ Maharajgunj Medical Campus, Institute of Medicine, Tribhuvan University Maharajgunj Nepal; ^4^ Nepal Health Frontiers, Tokha‐5 Kathmandu Nepal; ^5^ Department of Nephrology and Transplantation Medicine, Institute of Medicine Tribhuvan University Maharajgunj Nepal

**Keywords:** COVID‐19, CKD, hemodialysis, Moderna, Pfizer, vaccine

## Abstract

**Background and aims:**

Patients on maintenance dialysis are a high‐risk, immune‐compromised population with 15%–25% coronavirus disease (COVID‐19) mortality rate that has been underrepresented in COVID‐19 vaccination clinical trials. The aim of study was to review of those studies to determine the safety and efficacy of the COVID‐19 vaccination in chronic kidney disease (CKD) patients receiving maintenance hemodialysis systematically.

**Methods:**

The effectiveness was assessed by looking at the humoral and cellular responses. The humoral response is defined as de novo IgG‐ or IgA‐anti‐SpikeS1 antibody positivity. The establishment of de novo T‐cell immunity after immunization was used to measure cellular response. Adverse results were also reported of the included studies to analyze the safety of COVID‐19 vaccines. Eight previous works were included in our study.

**Results:**

Two doses of COVID‐19 vaccines were shown to be effective with seroconversion rate of humoral response ranging from 81% to 97% among eight studies. The T‐cell response was shown 67% and 100% in two studies. COVID‐19 vaccines did not have notable adverse events and hence can be considered safe.

**Conclusion:**

Although a single dosage has not shown to improve humoral immune response in most hemodialysis trials, a double dose has been reported to improve seroconversion rate and humoral immune response. Further research are required to observe if hemodialysis patients generate effective T‐cell responses.

## 1 INTRODUCTION

The coronavirus 19 disease (COVID‐19) caused by SARS‐CoV‐2 has led to loss of millions of lives across the globe since 2019. The susceptibility and complications are noted higher among immunocompromised groups. Chronic kidney disease (CKD) is one of the common immunocompromised states affecting larger population in the world. The global estimated prevalence of CKD is 13.4% (11.7%–15.1%), and patients with end‐stage kidney disease (ESKD) requiring renal replacement therapy is estimated between 4.902 and 7.083 million.^1^ CKD affects the morbidity and mortality through its effect on multiple organs of the body. Significant increase in number of patients of CKD and ESKD and death among these group due to poor access to renal replacement therapy has been leading to substantial financial burden for the developing countries.^1^

The rate of COVID‐19 infection in CKD patients is higher than the general population.^2^ ESKD patients under hemodialysis are highly susceptible to COVID‐19 due to other comorbidities, older age, immunocompromised status; frequent need of hospital visits for dialysis increase exposure and inability to maintain social distancing. The reasons for increased risk of symptomatic infection are impaired immunological status, chronic inflammation, high oxidative stress, accumulated uremic toxins, and endothelial dysfunction.^3^ The complications like acute respiratory distress syndrome (ARDS), acute cardiac injury, shock, and arrhythmias are higher in patients under dialysis, and mortality is reported higher 14% versus 4% in comparison to patient not infected with COVID‐19.^4^ Thus, to decrease the morbidity and mortality, it is essential to protect CKD patients from COVID‐19 and its complications. Apart from taking various precautions to prevent COVID‐19, vaccination helps to boost the immunity. However, the safety profile and the effectiveness of various COVID‐19 vaccines in CKD patients are not studied adequately.^5^

The aim of this study was to provide information on vaccination for COVID‐19 in CKD patients under maintenance hemodialysis for prevention of infection as well as decreasing the complications which ultimately improves the morbidity and mortality.

## 2 METHODS

### 2.1 Study identification

The article search was conducted in the online databases PubMed, Clinical trials.gov, google scholar, and EMBASE using the keywords “COVID,” “COVID‐19,” “SARS COV‐2,” “vaccine,” “Chronic kidney disease,” “CKD,” “ESKD,” “Hemodialysis,” “Dialysis,” “AstraZeneca,” “Vero cell,” “Pfizer,” and “Moderna” combined with “OR” and “AND” Boolean operators. Articles after 2019 were included in the study. Additional publications were found by searching the references list of the trials and articles included in the study. Following the retrieval of citations, titles and abstracts were assessed for potential eligible studies. Following an initial screening, the complete texts of potentially eligible papers were obtained for a more comprehensive review. Manual scanning of key articles and review papers was conducted to identify additional articles missed by the search strategy. We retrieved all references in all publications for further analysis. The protocol for reviews specified in Cochrane's handbook for systematic reviews of intervention^6^ and preferred reporting items for systematic reviews and meta‐analysis (PRISMA)^7^ was used to report the article (CRD42021282376). The first active search began on September 20, 2021, and the last was done on September 28, 2021.

### 2.2 Inclusion criteria

Studies were selected on the basis of the following criteria:

(I) Patients with CKD under hemodialysis.
(II) Any study design (randomized controlled trials with double‐blinded fold/observational studies/case‐control studies) on humans reporting clinical outcome of COVID‐19 vaccines.
(III) Patients who received any of the experimental COVID‐19 vaccines.

### 2.3 Exclusion criteria

Studies were excluded on the basis of the following criteria:

(I) Patients who did not receive any vaccine.
(II) Articles that were not available in English.
(III) All other forms of articles like case reports or letter to the editor were excluded.

### 2.4 Data extraction

Four authors (Sangam Shah, Kiran Paudel, Rajan Chamlagain, and Neha Mehta) extracted the data from the included studies and the studies and were recorded as follows:

A, Author; B, Year of study; C, Stage of the trial and CKD; D, Sample size; E, Study design; F, Country of study; G, Mean age; H, Gender; I, Efficacy measures; J, Adverse reactions.

For each of the investigations included, an adverse reaction was mentioned as per the inclusion criteria. Another author (Santosh Chhetri) double‐checked the extracted data, and disagreements were resolved by discussion among the authors.

### 2.5 Quality assessment

In the assessment, we looked at the following items: (1) whether the study objectives were clearly described; (2) whether the study period (start and end dates) was properly stated; (3) whether the patients selection criteria were described clearly; (4) whether the study was conducted in a multicenter setting; (5) if COVID‐19 vaccine treatment dose was stated; (6) whether the baseline equivalence groups were taken into account; (7) whether the primary outcome was defined before to the study; (8) whether the follow‐up period was long enough (months); (9) whether a clear hazard ratio (HR) with 95% confidence intervals (95% CI) was reported; and (10) Each study's limitations were taken into account. Quality assessment was not used as an exclusion criterion. The articles were graded based on the quality items utilized in each investigation (score range: 0–10).

### 2.6 Efficacy measurement

The following subheadings were used to examine the immunogenicity of patients.

i. **Humoral response:** De novo positivity of either IgG‐ or IgA‐anti‐SpikeS1 antibodies (without formation of virus‐specific NCP antibodies) after immunization indicates a positive humoral immune response.
ii. **T‐cell response:** Development of de novo T‐cell immunity induced by vaccination, clinical safety, and cellular immune response.

### 2.7 Data synthesis

The narrative summary contained all identified studies, as well as summary tables for characteristics. In addition, descriptive statistics were used to summarize the data. For continuous variables, we used the mean, and for dichotomous variables, we used frequencies and percentages.

## 3 RESULTS

### 3.1 Literature search

**Figure 1:**
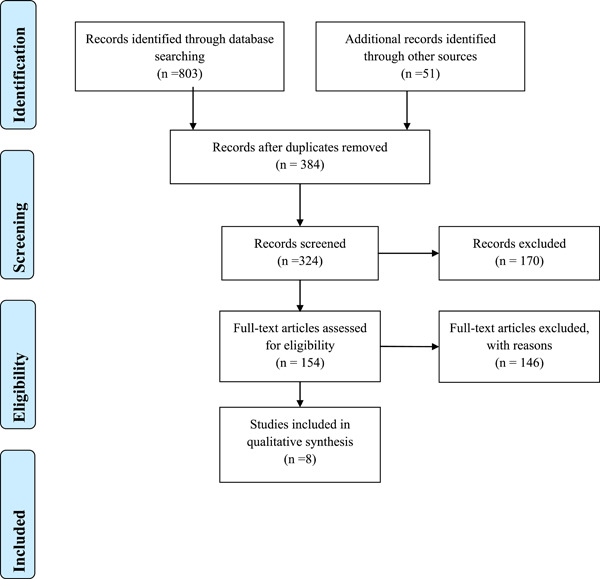
Preferred reporting items for systematic reviews and meta‐analysis guidelines for article identification and selection

The literature search resulted in 854 studies from PubMed, Google Scholar, EMBASE, and clinical trials.org. We screened total of 370 registered Covid ‐19 vaccine trials, 170 from Clinicaltrials.gov and 200 from WHO clinical trial database. After the complete screening process of titles, abstracts, and full texts, 846 studies did not meet the eligibility criteria. Finally, eight articles with different study designs that met the criteria were included in the review. A description of study selection is shown in the PRISMA flow diagram in Figure 1. The quality of the five included studies was fair with an average quality score of 6.75.^5^, ^6^, ^7^, ^8^ (Supporting Information: Table S1)

### 3.2 Literature identification

Two investigations were carried out in Germany and France, while one each was carried out in Austria, the United States, Canada, and Israel. Pfizer COVID‐19 vaccine was given to all eight trial participants, while Moderna COVID‐19 vaccine was given to participants in two studies. The studies by Bertrand et al and Lacson et al were retrospective while others were prospective observational and cohort studies. The study design, sample size, efficacy measures, adverse reactions are indicated in Table 1. There were 1826 hemodialysis patients in eight studies, with an average age of 71.1 years. The ratio of males to females was 1.78.

**Table 1:** Characteristics of included studies

SN no	Study	Sample size	Study design	Country of study	Name of vaccine	Mean age	Gender (%)	Body mass index	Adverse reaction
1	[10]	50	Observational cohort	Austria	Pfizer	67.6	M:34 F:16	NA	Local reaction, diarrhea, fatigue
2	[11]	1256	Prospective observational multicenter	Germany	Pfizer modern	67.6	M: 818 F: 438	27.5	Local reaction, fever, joint pain, chills
3	[12]	10	Retrospective, observational	France	Pfizer	71.2	M: 7 F: 3	NA	NA
4	[13]	186	Retrospective observational	USA	Pfizer modern	67.9	M:98 F:88	28.7	NA
5	[14]	154	Prospective	Canada	Pfizer	73	M:101 F:53	NA	Covid infection
6	[15]	56	Cohort	Israel	Pfizer	74	M:42 F:14	27.2	NA
7	[16]	78	Cohort	France	Pfizer	73.5	M:46 F:32	26.8	NA
8	Schrenzemeier et al (2021)	36	Cohort	Germany	Pfizer	74	M:25 F:11	NA	NA

Abbreviations: M, male; F, female; NA, not available.

### 3.3 Seroconversion

After reviewing the studies, we found that COVID‐19 vaccination in CKD patients were effective leading to seroconversion rate in 81%–97% of the vaccinated candidates at 4–8 weeks postvaccination interval following two doses of vaccine. The T‐cell response was 67% and 100% showed by the two studies which is mentioned in Table 2. However, the seroconversion rate was only 43% in one study which analyzed efficacy of a single dose of Pfizer vaccination.^8^ Similarly, the trials had the low antibody titer after 1st dose of vaccination and significant rise in the titer was noted after second dose. Thus, our review suggests that the two doses of vaccination is effective leading to high seroconversion rates and antibody titers. Although the studies showed seroconversion rates and adequate antibody titers, which were low in comparison to healthy controls.^9^ The nonresponders were found to be on long‐term immunosuppressants for the underlying comorbidities including diabetes.^8^ The studies showed no significant difference of efficacy between Pfizer and Moderna vaccine.

**Table 2:** Efficacy and seroconversion of included studies

SN no	Study	Efficacy measures	Seroconversion rate	Values
1	[10]	SARS Cov‐2 Antibody titre	42% after 1st dose and 97.9% after 2nd dose	After 1st dose: IgG concentration = 20.0 (11.7, 51.0) BAU/ml After 2nd dose: IgG concentration = 1075 (290.8, 1735) BAU/ml
2	[11]	Anti‐SARS Cov‐2 spike IgA and IgG antibody, Nucleocapsid IgG, T Cellular response	97% (Moderna) 87% (Pfizer)	**Humoral response** IgA‐Ab Spike S1 positive: At 3–4 weeks: 172(61.9%) At 8 weeks: 1083 (95.3%) IgG‐Ab Spike S1 positive: At 3–4 weeks: 140 (50.4%) At 8 weeks: 1074 (94.5%) **Cellular response** Interferon‐g release assay (IGRA) positive: At 3–4 weeks: 66 (44%) At 8 weeks: 30 (85.7%)
3	[12]	Anti‐SARS Cov‐2 spike antibody, T‐cell response	88.9% after 2nd dose 100% T‐cell responder	**Humoral response** After 3 weeks of 1st dose: 1 (11.1%) developed anti‐SARS‐CoV‐2 antibodies (*p* = 0.19). Antibody titers in responders were 178.9 AU/ml in HDP One month after 2nd dose, 8 (88.9%) developed anti‐SARS‐CoV‐2 antibodies (*p* < 0.0001) Median antibody titers in responders were 1052 AU/ml (IQR: 515–2689) **Cellular response** Three weeks after the 1st dose: 5 (55.6%) displayed significant number of spikes–reactive T cells (*p* = 0.06). In responders, median numbers of specific T cells were 208 SFC/106 CD3+T cells (IQR: 65–315) (*p* = 0.02). One month after the 2nd dose, a specific T cell response was detected in 9 HDP (100%) (*p* = 0.06). In responders, median numbers of spike–reactive T cells were 305 SFC/106 CD3 + T cells (IQR: 95–947) (*p* = 0.4)
4	[13]	Anti‐SARS Cov‐2 spike s1 specific IgG	88.7 (Pfizer) 94.4 (Moderna)	Spike‐Ab‐IgG was positive (≥2 U/L) in 165/186 (88.7%) without significant difference between BNT162b2/Pfizer (148/168) and mRNA‐1273/Moderna (17/18) vaccines at 88.1% versus 94.4%, *p* = 0.42. Nonresponders had mean spike‐Ab‐IgG titer of 0.4 ± 0.2 versus 18.2 ± 4.3 among responders (*p* < 0.001)
5	[14]	Anti‐RBD IgG	43%	Among those without previous SARS‐CoV‐2 infection, anti‐RBD IgG was undetectable at 4 weeks in 75 of 131 (57%, 95% CI: 47%–65%) patients receiving hemodialysis, compared with 1 of 20 (5%, 95% CI: 1%–23%) controls (*p* < 0.001). No patient with nondetectable levels at 4 weeks developed anti‐RBD IgG by 8 weeks. Results were similar in nonimmunosuppressed and younger individuals.
6	[15]	Anti‐SARS Cov‐2 spike s1 specific IgG	96%	The IgG levels in the dialysis group (median, 2900 AU/ml; interquartile range, 1128–5651) were significantly lower than in the control group (median, 7401; interquartile range, 3687–15,471). *U* = 1238; *p* = 0.001.
7	[16]	Anti‐SARS Cov‐2 spike s1 specific IgG	81%	Median antibody titer was 4.0 AU/ml (IQR, 1.85–12.2) at Day 14; 6.6 AU/ml (IQR 2.1–19.0) at Day 36; and 276 AU/ml (IQR 83.4–526.0) at Day 58
8	Schrenzemeier et al. (2021)	Anti‐SARS Cov‐2 spike antibody IgG and IgA, T‐cell response	88.9% IgG 91.67% IgA T‐cell response: 67.7%	**Humoral response** At 10 weeks after the 2nd dose, the proportion of dialysis patients reactive for anti‐SARS‐CoV‐2‐IgG decreased to 27/32 (84.37%, 95% CI: 66.46–94.10) The proportion of anti‐SARS‐CoV‐2 S1 IgA decreased from 33/36 (91.67%; 95% CI: 76.41–97.82) at Weeks 3–4 down to 19/32 (59.38; 95% CI: 40.79–75.78). **Cellular response** SARS‐CoV‐2‐specific T‐cell responses 3 weeks after second vaccination were detected in 21/31 vaccinated dialysis patients (67.7%, 95% CI: 48.53–82.68) compared to 42/44 (93.3%, 95% CI: 76.49–98.84) in controls of similar age

### 3.4 Humoral response

In the study by Zitt et al. 4 weeks after the Pfizer 42% of the patients developed a positive antibody response according to the assay‐specific cut‐off value of 33.8 BAU/ml with median (Q1, Q3) anti‐SARS‐CoV‐2 spike IgG concentration 20.0 (11.7, 51.0) BAU/ml. Four weeks after the second dose, the percentage of seropositive patients increased to 97.9% with a median (Q1, Q3) anti‐SARS‐CoV‐2 spike IgG concentration of 1075 (290.8, 1735) BAU/ml.^10^ Spike‐Ab‐IgG was positive (≥2 U/L) in 165/186 (88.7%) without significant difference between BNT162b2/Pfizer (148/168) and mRNA‐1273/Moderna (17/18) vaccines at 88.1% versus 94.4%, *p* = 0.42 in a study by Lacson et al.^13^ Nonresponders had mean spike‐Ab‐IgG titer of 0.4 ± 0.2 versus 18.2 ± 4.3 among responders (*p* < 0.001).^13^ The mean IgG levels in the dialysis group (median, 2900; interquartile range, 1128–5651) were significantly lower than those in the control group (median, 7401; interquartile range, 3687–15,471) and was statistically significant (U51238; *p* = 0.001) in a study by Grupper et al.^15^ Similar to this, patients undergoing hemodialysis had maximal response at Day 58, in which the median antibody titer was 4.0 AU/ml (IQR, 1.85–12.2) at Day 14; 6.6 AU/ml (IQR: 2.1–19.0) at Day 36; and 276 AU/ml (IQR: 83.4–526.0) at Day 58 in study by Danthu et al.^16^

In a study by Schrenzemeier et al., the antibody response rate of anti‐SARS‐CoV‐2‐IgG increased from 20/36 sera (55.56%; 95% CI: 38.29–71.67) at 1 week to 32/36 patients (88.9%; 95% CI: 73.0–96.4) within 3 weeks after the second dose and 33/36 sera (91.67%; 95% CI: 76.41–97.82) were also reactive for anti‐SARS‐CoV‐2‐IgA.^17^ But, after 10 weeks of vaccination the proportion of dialysis patients reactive for anti‐SARS‐CoV‐2‐IgG and anti‐SARS‐CoV‐2‐IgA decreased to 27/32 (84.37; 95% CI: 66.46–94.10) and 19/32 (59.38; 95% CI: 40.79–75.78), respectively.^17^ Three weeks after the first injection done, only one hemodialysis patient (11.1%) developed anti‐SARS‐CoV‐2 antibodies and antibody titers in responders were 178.9 AU/ml which increased 1 month after the second injection done, eight patients (88.9%) developed anti‐SARS‐CoV‐2 antibodies and median antibody titers in responders were 1052 AU/ml (IQR: 515–2689) in hemodialysis patient in a study by Bertrand et al.^12^

In the cohort of dialysis patients, seroconversion rates were 6 2% and 95% after 3–4 weeks and 8 weeks of first injection done respectively.^11^ In another study, receptor binding domain (RBD) antibodies were detected in 95% of seropositive dialysis patients at 8 weeks after the first dose of vaccine.^11^ Similarly, among patients without previous SARS‐CoV‐2 infection, those receiving hemodialysis exhibited significantly lower median anti‐RBD IgG levels at both 4 weeks (3 RLU, interquartile range [IQR] 3–8) and 8 weeks (3 RLU, IQR 3–9) after vaccination, compared with controls at 3 weeks (38 RLU, IQR 14–64, *p* < 0.001 for both comparisons), and compared with convalescent plasma from patients receiving hemodialysis who were survivors of COVID‐19 (77 RLU, IQR 18–199, *p* < 0.001) in Goupil et al.^14^ While among patients receiving hemodialysis, 75 of 131 (57%, 95% CI: 47%–65%) had anti‐RBD IgG levels that were nondetectable, compared with 1 of 20 controls (5%, 95% CI: 1%–23%, *p* < 0.001).^14^ By 8 weeks, none of the hemodialysis patients who had nondetectable antibodies at 4 weeks had produced detectable anti‐RBD. Anti‐RBD IgG levels were still considerably lower in patients receiving hemodialysis than in controls after 4 weeks, and they did not increase after 8 weeks.^14^

### 3.5 Cellular response

Cellular immune response was detected in 44% at T1 and 78% at T2 by interferon‐γ release assay (IGRA) in a study by Stumpf et al.^11^ Similarly in a study by Bertrand et al., 3 weeks after the first injection done and 1 month after the second injection done, five (55.6%) and nine (100%) hemodialysis patients (55.6%) displayed a significant number of spike–reactive T cells. In responders, median numbers of specific T cells were 208 SFC/106 CD3+T cells (IQR: 65–315) and 305 SFC/106 CD3+T cells (IQR: 95–947) after 1st and 2nd dose of vaccination among hemodialysis patients.^12^ Comparable to these findings there was a significant increase in the magnitude of vaccine‐reactive CD4+T‐cell at 8 weeks as compared to 3–4 weeks after double dose of Pfizer vaccine. SARS‐CoV‐2 specific T‐cells, were present 3 weeks after the second vaccination in 21/31 patients (67.74%; 95% CI: 48.53–82.68).^17^

### 3.6 Safety

Pain at the injection site within 7 days after the injection was the most commonly reported local reaction, occurring in 38% of the patients of mild degree after the first injection. After the second injection done, 29.2% of the patients reported mild, 2.1% moderate, and 2.1% severe local pain. Despite regular blood circuit anticoagulation with low‐molecular weight heparin and continuation of oral anticoagulation no hematoma occurred at the injection site, neither after the first nor the second vaccination.^10^ Local reaction, diarrhea, and fatigue were also recorded in few studies.^10^, ^11^ Seventeen patients (1.3%) of the dialysis cohort experienced COVID‐19 disease of whom five out of 17 (29%) died in study by Stumpf et al.^11^

## 4 DISCUSSION

The IgG antibody response to the spike protein following immunization with multiple COVID‐19 vaccines in patients on maintenance hemodialysis is described systematically in this study for the first time to the best of our knowledge. In comparison to the general population, patients on hemodialysis had a lower rate of humoral responses after vaccine injections done. It is known that dialysis patients have a lower immunization response. Patients on maintenance hemodialysis were not included in the crucial trial that demonstrated 95% protection against COVID‐19 infection after a two‐dose regimen of the BNT162b2 vaccination.^18^ Although the clinically significant antibody concentration cut‐off value for definitive sero protection is unknown, hemodialysis cohort's high seroconversion rate is remarkable when compared to other vaccine (other than COVID‐19 vaccine) in patients undergoing hemodialysis. Hemodialysis patients had a seroconversion rate ranging from 40% to 70% after receiving hepatitis B immunization.^19^, ^20^, ^21^, ^22^ In hemodialysis patients, vaccination against seasonal influenza and the 2009 pandemic influenza.

To avoid the interaction between SARS‐spike CoV‐2's protein and angiotensin‐converting enzyme 2, vaccine‐mediated immunity is developed (ACE‐2). Patients with renal illness should be chosen for COVID‐19 vaccination, and current evidence suggests that replication‐defective viral‐vectored and mRNA vaccines are safe for this population.

A virus H1N1 resulted in seroconversion rates ranging from 25% to 57%,^23^, ^24^, ^25^, ^26^, ^27^ with adjuvant vaccinations having greater response rates than non‐adjuvant vaccines.^28^ In comparison to these findings, the robust immunogenicity of the SARS‐CoV‐2 vaccine reported in the studies are encouraging, and it is expected that this will translate into the prevention of clinically relevant consequences such severe COVID‐19, hospitalization, and mortality. The study shows that the amount of anti‐HBs, which reflects the size of the humoral response to HBV vaccine, can be used to predict vaccine response for each patient. This may make it easier to tailor the anti‐COVID‐19 vaccine approach. These findings are consistent with prior research that found a reduced humoral response in kidney transplant recipients. For instance, Boyarsky et al. found that 54% of solid organ transplant recipients were immunized, whereas Benotmane et al. and Grupper et al. found that 48% and 37.5% of kidney transplant recipients, respectively, mounted a humoral response after two doses of mRNA vaccine.^15^, ^21^, ^22^ As a result, these findings are significantly different, with a lower immunization rate.

At Day 36, a study by Danthu et al. found that antibody production in the population followed a pattern similar to that of healthy people, with a similar rate of responders.^16^ However, as measured by antibody levels, the humoral response in patients on hemodialysis was delayed, heterogeneous, and of lower intensity. Grupper et al.^15^ found that patients on hemodialysis have a lower humoral response. The hemodialysis patients' humoral reaction was related to age, serum albumin, and number of hemodialysis (Kt/V). These factors are well‐known to be linked to immunological state, and hence have the potential to alter humoral responses particularly in uremic individuals.^29^, ^30^ Our findings support the recently published high antibody response rates of 90%–96% in three Israeli dialysis cohorts,^15^, ^31^, ^32^ and show a greater vaccine response when compared to 56 SARS‐CoV‐2 infection‐free hemodialysis patients in France (82%).^35^

Patients with kidney failure (KF) who are on chronic dialysis have a lower number of B and T cells, which may contribute to the disparities in comparison with non‐KF patients.^34^, ^35^, ^36^ Furthermore, reduced uremic toxin clearance, systemic inflammation, and starvation may all contribute to decreased immunogenicity. The extensive variety of antigenic cues offered by the entire SARS‐CoV‐2‐ in comparison to the vaccine's spike‐protein may explain the differential in immune response when compared to spontaneous infection. In fact, our findings correlate with other studies which have shown that two additional proteins, membrane, and nucleocapsid, have antigenic characteristics. Furthermore, the immunogenic dominance of SARS‐CoV‐2 proteins varies by patient, with some patients having very few or no spike‐reactive T cells but high frequencies of M‐ or N‐reactive T cells.^37^

Hemodialysis patients should be prioritized for quick vaccination due to their delayed antibody response, and the period between the first and second doses should not be increased. The fact that mRNA‐based vaccinations have a high prevalence of adverse effects and strong antibody responses suggests that they may be more immunogenic than traditional vaccines. In a way comparable to natural infection, mRNA vaccines have been demonstrated to trigger the development of neutralizing antibodies targeting the same epitopes.^38^ As indicated by Grupper et al.^15^ and Agur et al.^31^ and corroborated in our investigation, age is a well‐known primary determinant of vaccination response in hemodialysis patients, with older age being linked with a weaker antibody concentration.

Potential immunosuppression and acute hospitalization during the vaccine series, particularly in the situation of hypoalbuminemia and uremic inflammation, have face validity in influencing immune response.^39^, ^40^, ^41^ In contrast to healthy people, who can presumably safely delay the second dose, most hemodialysis patients are unlikely to be able to do so.^42^ Anti‐RBD IgG does not appear to induce immunity against SARS CoV‐2 infection, but it is a useful surrogate because anti‐RBD IgG correlates highly with viral neutralization,^43^ Fc‐mediated effector activities, and cellular responses.^44^ Furthermore, all COVID‐19 survivors in study by Goupil et al had an anti‐RBD response 4–16 weeks after infection.^14^ The possibility that vaccinated hemodialysis patients with undetectable anti‐RBD IgG develops protective cellular immune responses cannot be ruled out, but this is unlikely given that neutralization, Fc function, and SARS‐CoV‐2–specific T‐cell responses have only been observed in people who elicited RBD‐specific antibodies in previous studies.^44^, ^45^ The absence of clinical COVID‐19 following vaccination is the most conclusive evidence of vaccine effectiveness.

SARS‐CoV‐2‐S1 IgG antibody levels in every seventh dialysis patient maintained or fell below the threshold for positive titers during the 10‐week longitudinal follow‐up period after the second dose of Tozinameran vaccination, as shown by pairwise comparison of anti‐SARS‐CoV‐2 IgG responses.^17^ The presence of SARS‐CoV‐2‐S1 IgA in dialysis patients' serum following mRNA vaccination is a noteworthy finding, as mucosal IgA is important for respiratory infection defense.^46^ It is uncertain whether IgA measured in serum after vaccination is related to mucosal immunity, and more research is needed, particularly longitudinal comparisons with nondialysis groups. After SARS‐CoV‐2 infection, specific IgA antibodies against SARS‐CoV‐2 have been demonstrated to rapidly degrade, and this may be even more pronounced in vaccinated persons.^47^

Despite intramuscular injection during the hemodialysis session with full low‐molecular weight heparin anticoagulation, no substantial local hematoma developed. As a result, intramuscular immunization can be safely done in hemodialysis patients during the last 30 min of their treatment session. This allows for proper postvaccination observation and vital sign monitoring, with no changes to the patients' logistics or hemodialysis prescription required. This technique is supported by the CDC's overall best practice guidelines for immunization in people with bleeding disorders or those taking warfarin in the general population.^48^

## 5 CONCLUSION

The effectiveness of SARS‐CoV‐2 vaccine in hemodialysis patients requires further investigation. A single dose has not shown to improve humoral immune response in most studies with hemodialysis patients; however, a double dose has been reported to improve seroconversion rate and humoral immune response. More research is needed to see if hemodialysis patients generate effective T‐cell responses. Also, there are no notable adverse effects of vaccines in hemodialysis patients. To date, patients requiring hemodialysis should get the second dose of Pfizer and Moderna vaccine at the prescribed 3‐week interval, and strict SARS‐CoV‐2 infection prevention and control measures should be continued in hemodialysis units until vaccine efficacy is completely determined.

## AUTHOR CONTRIBUTIONS

**Neha Mehata**: Conceptualization; data curation; formal analysis; investigation; methodology; writing—original draft; writing—review and editing. **Sangam Shah**: Conceptualization; data curation; formal analysis; investigation; methodology; writing—original draft; writing—review and editing. **Kiran Paudel**: Conceptualization; formal analysis; methodology; visualization; writing—original draft; writing—review and editing. **Rajan Chamlagain**: Data curation; investigation; methodology; project administration; writing—review and editing. **Santosh Chhetri**: Investigation; methodology; supervision; validation; writing—original draft; writing—review and editing.

## CONFLICT OF INTEREST

The authors declare no conflict of interest.

## TRANSPARENCY STATEMENT

All authors have read and approved the final version of the manuscript. The first and corresponding author had full access to all of the data in this study and take complete responsibility for the integrity of the data and the accuracy of the data analysis. The lead author (Dr. Neha Mehata) affirms that this manuscript is an honest, accurate, and transparent account of the study being reported; that no important aspects of the study have been omitted; and that any discrepancies from the study as planned (and, if relevant, registered) have been explained.

## Supporting information

Supporting information.

## Data Availability

All the required data are included in the manuscript.
