# Supplementary material for: Safety and efficacy of coronavirus disease‐19 vaccines in chronic kidney disease patients under maintenance hemodialysis: A systematic review
Source: Health Sci Rep. 2022 Jun 16;5(4):e700. doi: 10.1002/hsr2.700 (PMC9203992; doi:10.1002/hsr2.700)
Supplement: Supplementary file 1 — Supporting information. [file HSR2-5-0-s001.docx]

| Studies  Criteria | Zitt et al, 2021 | Stumpf et al, 2021 | Bertrand et al, 2021 | Lacson et al, 2021 | Goupil et al, 2021 | Grupper et al, 2021 | Danthu et al, 2021 | Schrenzemeier et al, 2021 |
| --- | --- | --- | --- | --- | --- | --- | --- | --- |
| Clarity of the study objectives | Yes | Yes | Yes | Yes | Yes | Yes | Yes | Yes |
| Whether the study period (start date and end date) was stated clearly | Yes | Yes | Yes | Yes | Yes | Yes | Yes | Yes |
| Whether the description of the patient selection criteria was clear or not | Yes | Yes | No | No | Yes | No | Yes | Yes |
| the stated COVID-19 vaccine treatment dose (1 or 2 dose) | Yes | Yes | Yes | Yes | Yes | Yes | Yes | Yes |
| study was done in multi center or not | No | Yes | No | No | No | No | No | No |
| Whether the baseline equivalence groups were clearly considered | Yes | No | Yes | Yes | Yes | Yes | Yes | Yes |
| whether the primary outcome was defined before to the study | Yes | Yes | Yes | Yes | Yes | Yes | Yes | Yes |
| If the follow-up period was long enough (at least two months) | No | No | No | No | No | No | Yes | Yes |
| whether a clear hazard ratio (HR) with 95% confdence intervals (95% CI) was stated | No | No | No | No | No | No | No | No |
| The limitations of each study were considered | Yes | Yes | No | Yes | Yes | Yes | Yes | Yes |
| Overall score | 7 | 7 | 5 | 6 | 7 | 6 | 8 | 8 |

Mean: 6.75

Supplemental table 1: Quality assessment of the included studies
